# Multi-omic prediction of incident type 2 diabetes

**DOI:** 10.1007/s00125-023-06027-x

**Published:** 2023-10-27

**Authors:** Julia Carrasco-Zanini, Maik Pietzner, Eleanor Wheeler, Nicola D. Kerrison, Claudia Langenberg, Nicholas J. Wareham

**Affiliations:** 1 MRC Epidemiology Unit, School of Clinical Medicine, University of Cambridge, Institute of Metabolic Science, Cambridge, UK; 2 Computational Medicine, Berlin Institute of Health at Charité-Universitätsmedizin Berlin, Berlin, Germany; 3 Precision Healthcare University Research Institute, Queen Mary University of London, London, UK

**Keywords:** Biomarkers, Genomics, Metabolomics, Prediction models, Proteomics, Type 2 diabetes

## Abstract

**Aims/hypothesis:**

The identification of people who are at high risk of developing type 2 diabetes is a key part of population-level prevention strategies. Previous studies have evaluated the predictive utility of omics measurements, such as metabolites, proteins or polygenic scores, but have considered these separately. The improvement that combined omics biomarkers can provide over and above current clinical standard models is unclear. The aim of this study was to test the predictive performance of genome, proteome, metabolome and clinical biomarkers when added to established clinical prediction models for type 2 diabetes.

**Methods:**

We developed sparse interpretable prediction models in a prospective, nested type 2 diabetes case-cohort study (*N*=1105, incident type 2 diabetes cases=375) with 10,792 person-years of follow-up, selecting from 5759 features across the genome, proteome, metabolome and clinical biomarkers using least absolute shrinkage and selection operator (LASSO) regression. We compared the predictive performance of omics-derived predictors with a clinical model including the variables from the Cambridge Diabetes Risk Score and HbA_1c_.

**Results:**

Among single omics prediction models that did not include clinical risk factors, the top ten proteins alone achieved the highest performance (concordance index [C index]=0.82 [95% CI 0.75, 0.88]), suggesting the proteome as the most informative single omic layer in the absence of clinical information. However, the largest improvement in prediction of type 2 diabetes incidence over and above the clinical model was achieved by the top ten features across several omic layers (C index=0.87 [95% CI 0.82, 0.92], Δ C index=0.05, *p*=0.045). This improvement by the top ten omic features was also evident in individuals with HbA_1c_ <42 mmol/mol (6.0%), the threshold for prediabetes (C index=0.84 [95% CI 0.77, 0.90], Δ C index=0.07, *p*=0.03), the group in whom prediction would be most useful since they are not targeted for preventative interventions by current clinical guidelines. In this subgroup, the type 2 diabetes polygenic risk score was the major contributor to the improvement in prediction, and achieved a comparable improvement in performance when added onto the clinical model alone (C index=0.83 [95% CI 0.75, 0.90], Δ C index=0.06, *p*=0.002). However, compared with those with prediabetes, individuals at high polygenic risk in this group had only around half the absolute risk for type 2 diabetes over a 20 year period.

**Conclusions/interpretation:**

Omic approaches provided marginal improvements in prediction of incident type 2 diabetes. However, while a polygenic risk score does improve prediction in people with an HbA_1c_ in the normoglycaemic range, the group in whom prediction would be most useful, even individuals with a high polygenic burden in that subgroup had a low absolute type 2 diabetes risk. This suggests a limited feasibility of implementing targeted population-based genetic screening for preventative interventions.

**Graphical Abstract:**

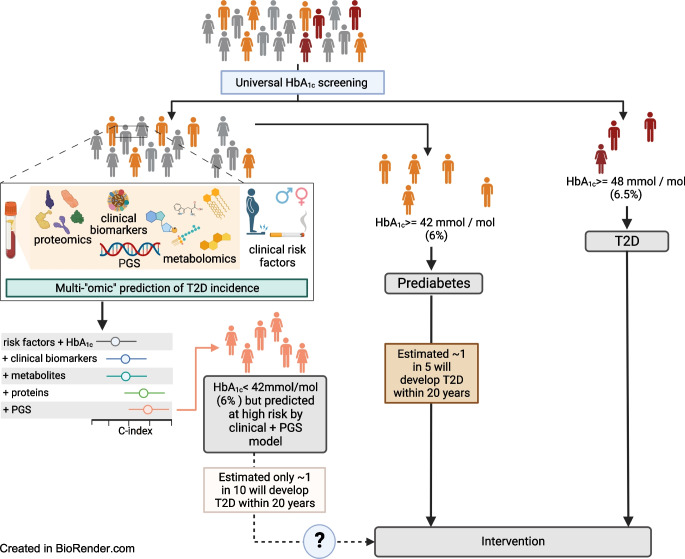

**Supplementary Information:**

The online version of this article (10.1007/s00125-023-06027-x) contains peer-reviewed but unedited supplementary material.


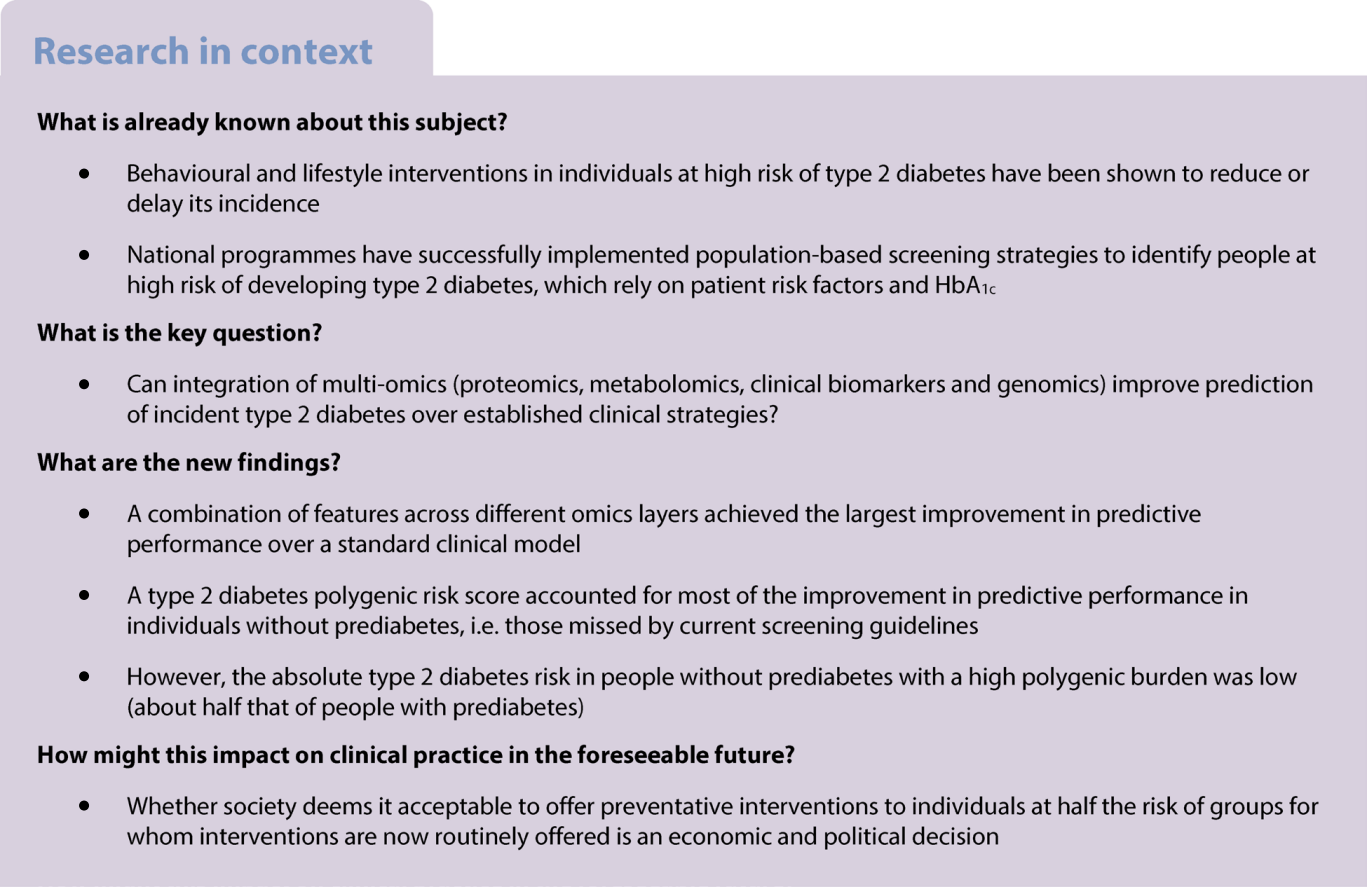


## Introduction

Type 2 diabetes is predicted to affect 10.9% of the world’s population by 2045 [1], motivating the development of early risk assessment models and national screening programmes [2]. Changes in glucose metabolism are detectable years before type 2 diabetes onset, and yet some individuals remain undiagnosed for many years [3]. Screening for hyperglycaemia can detect people with previously undiagnosed diabetes and early treatment can reduce the risk of complications [4]. Measurement of glucose levels and HbA_1c_ can also identify individuals at high risk of diabetes, and trials, predominantly in people with impaired glucose tolerance, have demonstrated the benefits of early behavioural and pharmacological intervention [5]. The identification of risk factors and biomarkers that can identify people at high risk of type 2 diabetes has been an active area of research. Several risk scores based on readily available patient-derived information have been developed and tested, achieving already good predictive performance (concordance index [C index] ranging from 0.73 to 0.81) [6]. Additional inclusion of blood-based clinical biomarkers (such as HbA_1c_, glucose, lipid profiles, uric acid and γ-glutamyl transferase [GGT]) has been shown to provide improvements in C indices of up to 0.9 [7], mostly provided by diagnostic markers: HbA_1c_ and glucose measurements. However, systematic investigation of the added predictive performance provided by these biomarkers in the subset of individuals that are not classified as high risk by the current clinical guidelines has not been reported so far.

Genome-wide association studies (GWAS) have identified hundreds of type 2 diabetes genetic risk variants [8, 9]. However, genome-wide polygenic risk scores (PGSs) have not been shown to provide clinically meaningful improvements in predictive performance over and above what can be achieved with simple clinical models [10, 11]. Recent technological advancements in other broad capture omic technologies now enable systematic investigation of the individual and joint values of thousands of easily accessible blood molecules from different biological layers in a hypothesis-free manner. Early omic studies have tested the improvement in performance by single layers of biological information in isolation [12, 13] or by using complex signatures that include hundreds of features [14, 15], restricting the potential for clinical translation.

In this prospective study, comprising more than 300 deeply phenotyped incident type 2 diabetes cases, we systematically tested whether integration of biological information across the genome, the circulating metabolome and proteome, and a wide range of clinical biomarkers could provide improved predictive performances over and above what can be achieved by a currently available clinical model.

## Methods

### Study design

The EPIC-Norfolk study is a cohort of 25,639 men and women aged 40–79 years at baseline in 1993–1997, which has previously been described in detail [16]. The EPIC-Norfolk study was approved by the Norfolk Research Ethics Committee (ref. 05/Q0101/191); all participants gave their informed written consent before entering the study.

**Fig. 1:**
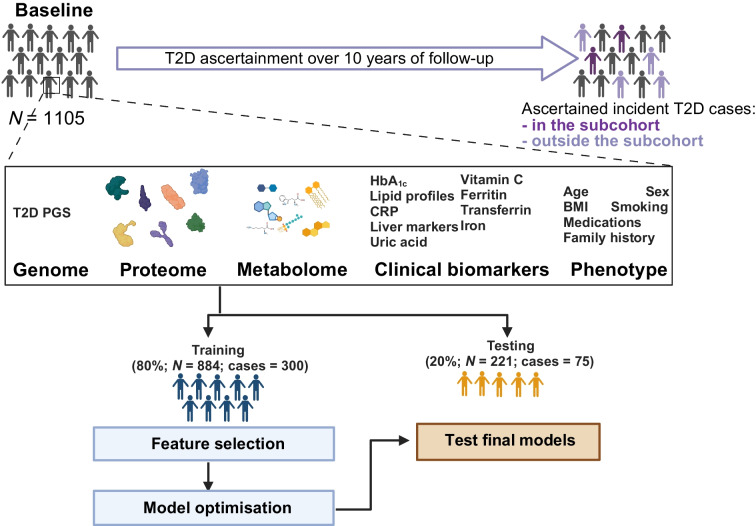
Study design. We designed a case-cohort (*N*=1105) for incident type 2 diabetes within the EPIC-Norfolk study. Genotyping, proteomics (SomaScan v4), metabolomics (Metabolon Discovery HD4) and biomarker profiling were done in samples from the baseline assessment. T2D, type 2 diabetes. Created with BioRender.com

Here, we designed a prospective type 2 diabetes case-cohort (Fig. 1) based on all individuals with no evidence of prevalent diabetes at baseline and available stored blood samples [17]. We ascertained and verified all individuals who developed incident type 2 diabetes over 10 years (follow-up was censored at date of type 2 diabetes diagnosis up to 31 December 2007) based on self-report, doctor-diagnosed diabetes, diabetes drug use or evidence of diabetes after baseline with a date of diagnosis prior to the date of baseline visit, as described previously [18]. We randomly selected 875 individuals from eligible participants as a control cohort. Participants with evidence of prevalent diagnosed diabetes, or those with prevalent diabetes but undiagnosed at baseline, HbA_1c _≥48 mmol/mol (6.5%), were excluded. We further used the WHO threshold to define prediabetes according to HbA_1c_ levels as ≥42 mmol/mol (6.0%) and <48 mmol/mol (6.5%).

### Type 2 diabetes PGS

Genome-wide genotyping was performed using the Affymetrix UK Biobank Axiom Array (Thermo Fisher Scientific, Santa Clara, USA), with imputation to the Haplotype Reference Consortium r1.0 and the UK10K plus 1000 Genomes phase 3 reference panels. We generated a genome-wide PGS for type 2 diabetes using LDpred2 [19] (bigsnpr R package v1.8.8 [20]), which has been shown to outperform traditional pruning and thresholding methods. Briefly, this method uses a Bayesian approach that relies on genome-wide summary statistics and linkage disequilibrium (LD) information from an external reference panel to infer posterior mean effect size for each of the variants. Quality control for individual-level genotype data was performed using PLINK v1.9 (https://www.cog-genomics.org/plink/1.9) [21, 22]. We removed strand ambiguous variants with a minor allele frequency (MAF) <1%, Hardy–Weinberg equilibrium *p*<1×10^−6^ or a missing rate >1%. Individuals with a genotype missing rate >10% or those with a first- or second-degree relative in the sample were removed (*N*=17). We further restricted PGS generation to HapMap3 variants as suggested by the LDpred2 authors. PGSs were generated using the infinitesimal model option based on 721,911 variants. Summary statistics were obtained from the largest European meta-analysis including 228,499 type 2 diabetes cases [9].

### Omics profiling

For the individuals included in the case-cohort, we used citrate plasma samples that had been stored in liquid nitrogen since the baseline assessment, which took place between 1993 and 1997, for proteomics profiling using the SomaScan v4 assay (SomaLogic, Boulder, USA). Assay details have been described previously [14]. Briefly, this assay uses 4979 aptamer reagents to target 4775 unique proteins. Normalisation was performed by SomaLogic using adaptive normalisation by maximum likelihood (ANML). For all analyses, protein relative fluorescence intensities were log_10_-transformed and scaled to have a mean of zero and variance of 1.

Untargeted metabolomics profiling was done in samples from the baseline visit for individuals included in the case-cohort using the Metabolon Discovery HD4 platform, as previously described [23] (Metabolon, Durham, USA). We kept data from only 762 metabolites with no more than 50% of missing values overall and no more than 50% of missing values in incident type 2 diabetes cases. Missing values were imputed using the missForest v1.4 R package [24]. Metabolites were natural log-transformed and scaled. We further measured 17 biomarkers and again imputed missing values with the missForest R package. Transferrin, albumin, alkaline phosphatase (ALP), alanine aminotransferase (ALT), apolipoprotein (Apo)A1, ApoB, aspartate aminotransferase (AST), C-reactive protein (CRP), ferritin, GGT, iron and uric acid were measured by standard immunoassays (Olympus AU640 Analyzer). Total cholesterol, triglycerides and HDL-cholesterol were analysed on an RA-1000 (Bayer Diagnostics, Basingstoke, UK). LDL-cholesterol was calculated with the Friedwald formula [25], except for when triglyceride levels were >4 mmol/l. Vitamin C levels were measured with a fluorometric assay from plasma that was stabilised using metaphosphoric acid [26]. HbA_1c_ was measured as part of the EPIC-Interact project using a Tosoh (HLC-723G8) assay on a Tosoh G8 analyser.

### Derivation and validation of predictive models

We excluded participants with missing genotype data, HbA_1c_ or information on variables included in the Cambridge Diabetes Risk Score [27], leaving 1105 participants (10,792 person-years of follow-up) for analyses (375 incident type 2 diabetes cases) (electronic supplementary material [ESM] Fig. 1a). To have an independent internal validation set, completely blinded to any previous feature selection and hyperparameter tuning steps, we divided individuals into a training set (80%, *N*=884) and a testing set (20%, *N*=221). We trained models using regularised Cox regression, including a patient-derived information model, which is based on variables used for the Cambridge Diabetes Risk Score (age, self-reported sex, family history of diabetes, smoking status, prescription of antihypertensive medication and BMI) [27], and a standard clinical model (including the variables from the Cambridge Diabetes Risk Score and HbA_1c_). We refitted a model using variables from the Cambridge Diabetes Risk Score to find optimal weights in EPIC-Norfolk and to enable a fair comparison among all models.

For omic predictors, we first performed feature selection in the training set. Feature selection was carried out by least absolute shrinkage and selection operator (LASSO) to identify the top predictors among the proteome, the metabolome and 17 clinical biomarkers. A nested tenfold cross-validation (inner loop to determine regularisation parameter, ʎ) was done over 100 subsamples, taking 80% of the training set (outer loop). Within each omic layer, we ranked each feature based on an absolute weighted sum of the number of times it was included in the final model from each of the 100 subsamples and selected the ten features with the highest rankings separately for each omic layer. We additionally performed feature selection across all omic layers (including all proteins and metabolites and the type 2 diabetes PGS) to identify the top ten omic predictors. We implemented this workflow using the R packages caret v6.0-89 [28] and glmnet v4.1 [29]. Features selected were taken forward for parameter optimisation by tenfold cross-validation of the model by regularised Cox regression (incorporating Prentice weights to account for the case-cohort design [30, 31]) in the entire training set. For each of the omic layers, top predictors were optimised alone or on top of the standard clinical model, for which variables were forced to be kept in the models.

Performance of the classification models was evaluated in the internal independent validation set, which was never used for training and optimisation. The prediction models’ discriminatory power was assessed by computing the C index over 1000 bootstrap samples of the test set. We further tested the model’s performance by stratifying the test set into participants with (*N*=176) and without prediabetes (defined as HbA_1c_ ≥42 mmol/mol [6.0%], *N*=46). Performance of PGS-only models was tested using simple Cox proportional hazards models (Prentice-weighted), using an analogous bootstrapping framework. To determine whether the addition of omics improved performance over the clinical model, we estimated one-sided *p* values for the difference in the mean C indices (from bootstrapping). This was calculated as the number of times the difference (between the C indices from clinical + omics and the clinical model) was lower than zero divided by 1000 (that is, the number of bootstrap C indices). For each of the omic layers, we calculated the net reclassification improvement (NRI) when added on top of the clinical model in the test set (all and stratifying by the HbA_1c_ threshold for prediabetes) using the R package nricens v1.6 [32].

### Cumulative type 2 diabetes incidence

To be able to compute absolute risk estimates, we leveraged genotype information available for participants from the entire EPIC-Norfolk study. Linkage with hospitalisation and death registry data was done using UK National Health Service (NHS) numbers through NHS Digital. Vital status was ascertained for the entire EPIC-Norfolk cohort and death certificates were coded by trained nosologists according to ICD-10. For type 2 diabetes definitions, participants were identified as having an event if the corresponding ICD-10 code was registered on the death certificate (as the underlying cause of death or as a contributing factor) or as the cause of hospitalisation. Since the long-term follow-up of EPIC-Norfolk included a period in which coding shifted from ICD-9 to ICD-10, codes were consolidated. We computed the predicted risk for incident type 2 diabetes using the clinical + PGS model in the entire EPIC-Norfolk cohort, excluding individuals with HbA_1c_ levels above the threshold for diabetes (≥48 mmol/mol [6.5%]), with incomplete data for the variables included in this model or who were included in the training set from the case-cohort study (*N*=9009, ESM Fig. 1b). We stratified participants into those with prediabetes according to their HbA_1c_ levels (≥42 mmol/mol [6.5%]) and quartiles of predicted risk according to the clinical + PGS model among individuals with normoglycaemia. We estimated the cumulative incidence of type 2 diabetes over a 20 year follow-up period in these five strata as 1 minus the Kaplan–Meier estimate of the survivor function.

## Results

Baseline characteristics are presented in ESM Table 1. Incident type 2 diabetes cases were on average older, more likely to be men and presented with higher BMI and HbA_1c_ levels compared with cohort control participants.

### Comparison between the predictive performance of sparse omics signatures and standard clinical models

The Cambridge Diabetes Risk Score model achieved a C index of 0.76 (95% CI 0.69, 0.82). The top ten proteins achieved the highest predictive performance compared with the Cambridge Diabetes Risk Score model (C index 0.82 [0.75, 0.88], Δ C index=0.06, *p*=0.042) out of all single omic predictors, followed by clinical biomarkers (C index 0.78 [0.72, 0.85], Δ C index=0.02, *p*=0.29), metabolites (C index 0.78 [0.71, 0.84], Δ C index=0.02, *p*=0.28) and the type 2 diabetes PGS (C index 0.69 [0.60, 0.76], Δ C index=−0.07, *p*=0.1). Besides proteins, the top ten combined omic features (C index 0.86 [0.80, 0.91]) had a significantly higher predictive performance compared with the basic Cambridge Diabetes Risk Score model (Δ C index=0.10, *p*=0.004).

**Fig. 2:**
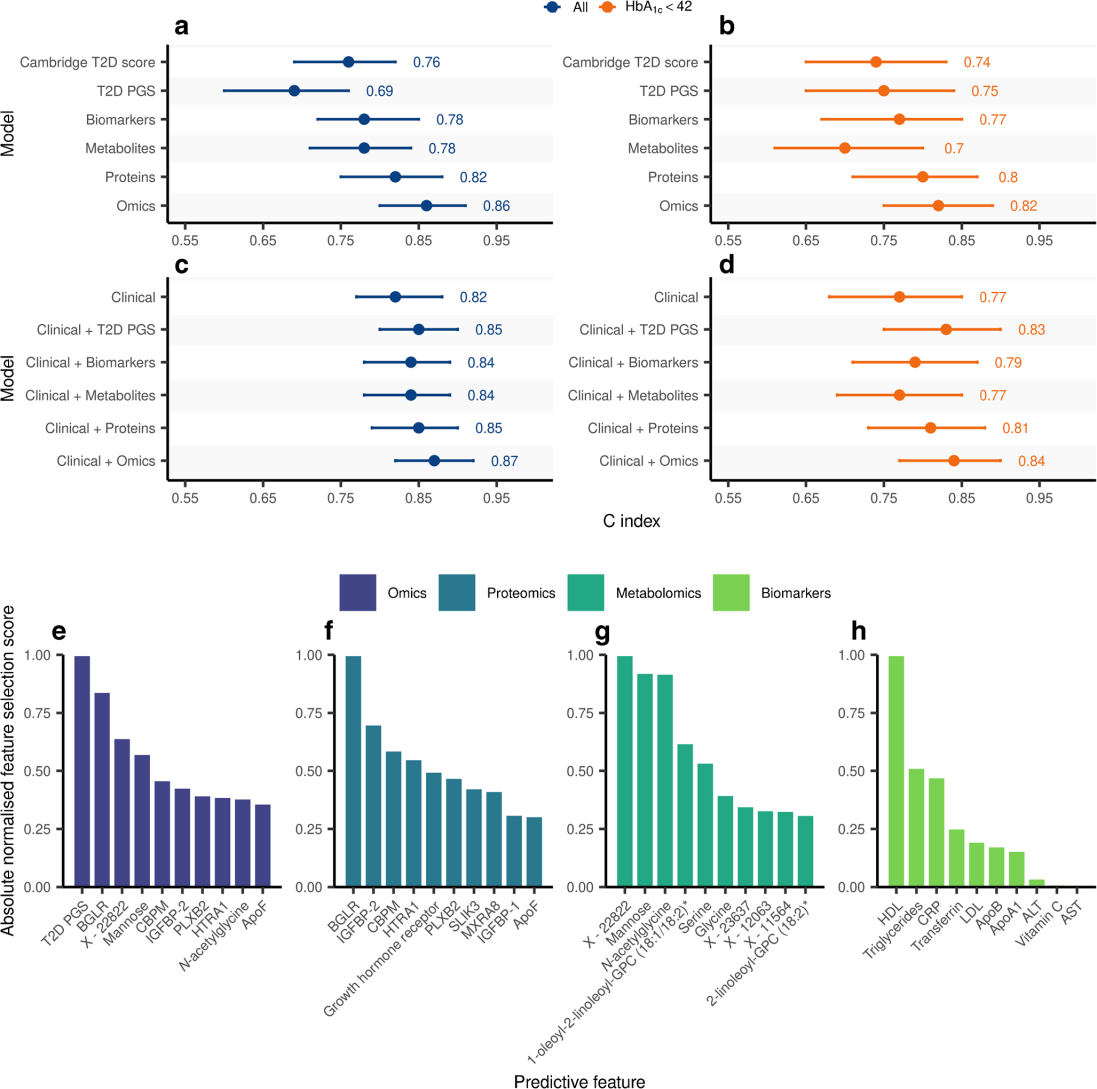
Multi-omic prediction of type 2 diabetes incidence. (**a**–**d**) C index of the prediction models in all individuals from the internal validation set or by stratifying into individuals with prediabetes (HbA_1c_ ≥42 mmol/mol [6.5%], *n*=45) and individuals with normoglycaemia (HbA_1c_ <42 mmol/mol [6.5%], *n*=171). The 95% CI from bootstrapping is shown. (**e**–**h**) Top ten features selected from each of the omic layers. Selection scores are shown normalised to the feature with the highest score for interpretability. 2-linoleoyl-GPC, 2-linoleoyl-glyceroposphocholin; MXRA8, matrix remodelling associated protein 8; 1-oleoyl-2-linoleoyl-GPC, 1-oleoyl-2-linoleoyl-glyceroposphocholin; SLIK3, SLIT and NTRK like family member 3; T2D, type 2 diabetes

We derived a clinical benchmark model by adding HbA_1c_ to the Cambridge Diabetes Risk Score, which performed significantly better (C index=0.82 [0.77, 0.88], Δ C index=0.06, *p*=0.002). Beyond HbA_1c_, only the top ten omic features (C index 0.87 [0.82, 0.92]) significantly improved the C index over the standard clinical benchmark (Δ C index=0.05, *p*=0.045) (Fig. 2a). However, the largest NRI over the clinical model (that is, the Cambridge Diabetes Risk Score + HbA_1c_) was provided by the top ten proteins (NRI=0.19), followed by the top ten omic features (NRI=0.14, ESM Table 2). Among the top ten omics predictors were several markers also selected among the top ten proteins, such as β-glucuronidase (BGLR), carboxypeptidase M (CBPM), insulin-like growth factor binding protein (IGFBP)-2, plexin B2 (PLXB2), serine protease HTRA1 and ApoF; or the top ten metabolites, including mannose, *N*-acetylglycine and a metabolite of unknown identity (X-22822). We provide all model coefficients in ESM Table 3.

### Improvements in prediction over and above a standard clinical model in individuals without prediabetes

According to current clinical guidelines in England, individuals with HbA_1c_ levels above the prediabetic threshold (≥42 mmol/mol [6.0%]) would be offered a referral to a behavioural intervention programme [33]. As clinical action differs depending on whether or not an individual is deemed to have prediabetes, we therefore assessed the predictive performance provided by the different omic layers by stratifying the internal validation test set into individuals with prediabetes and with normoglycaemia according to their HbA_1c_ measurements. In the group of individuals without prediabetes (HbA_1c_ <42 mmol/mol [6.0%]), in whom prediction would be most relevant, the Cambridge Diabetes Risk Score model (C index=0.74 [0.65, 0.83]) was not improved by the addition of HbA_1c_ (C index=0.77 [0.68, 0.85], Δ C index=0.03, *p*=0.20). This is well in agreement with the selection of the HbA_1c_ threshold to define prediabetes. The maximum improvement over the clinical model in this subgroup was achieved by adding the top ten omic predictors (C index=0.84 [0.77, 0.90], Δ C index=0.07, *p*=0.03), which resulted in the highest NRI (0.27), mainly attributed to correct reclassification of cases (ESM Table 4). Stepwise addition of omic biomarkers did not provide any further significant improvements (ESM Table 5). Among the top ten predictor omic features, the type 2 diabetes PGS had the largest weight and was the most frequently selected in different iterations (Fig. 2b). The type 2 diabetes PGS alone provided a comparable improvement over the clinical model (C index=0.83 [0.75, 0.90], Δ C index=0.06, *p*=0.002) to the top ten omic features. However, we note the relative difference in information content when comparing these predictors, as the type 2 diabetes PGS includes genome-wide information, compared with information on only ten features for the other omic predictors. In the subgroup of people with prediabetes, none of the omic predictors improved the performance over and above the clinical model (ESM Table 6), although the small sample size in this subgroup was insufficient to draw robust conclusions. However, we note that prediction in this subgroup is less relevant in clinical practice, as these individuals would be referred for preventative interventions.

### Absolute type 2 diabetes risk in individuals without prediabetes with a high polygenic burden

**Fig. 3:**
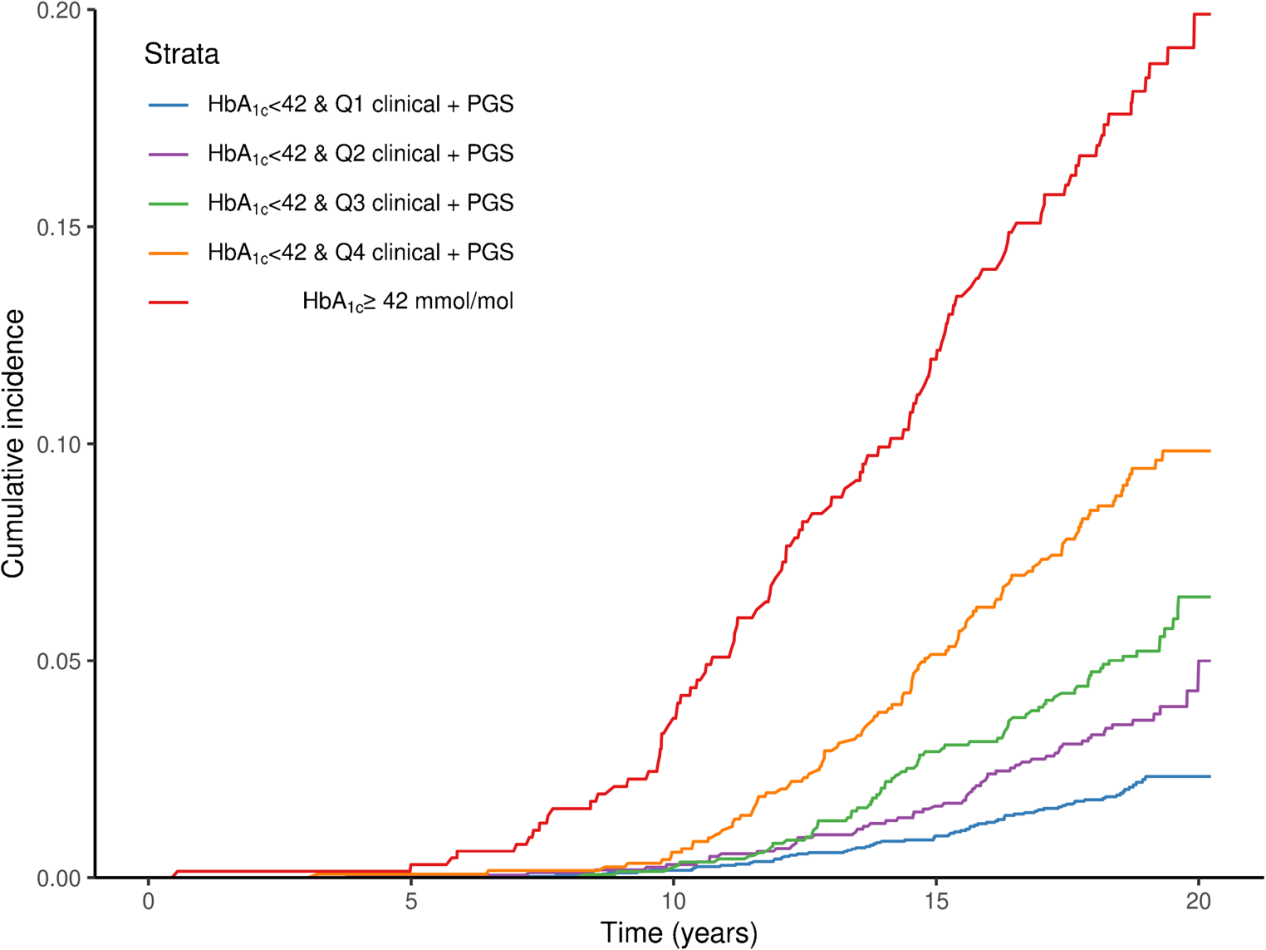
Cumulative incidence of type 2 diabetes over 20 years in individuals with prediabetes compared with quartiles of clinical + polygenic risk. The clinical + PGS model was used to divide individuals with normoglycaemia into quartiles according to predicted risk and to estimate the cumulative incidence among these groups compared with the cumulative incidence in individuals with prediabetes

We next sought to quantify the absolute risk for type 2 diabetes among individuals with prediabetes compared with those without prediabetes, but at high polygenic risk, in the entire EPIC-Norfolk cohort with complete data (*N*=9009, ESM Table 7) (Methods). The cumulative incidence at 5, 10, 15 and 20 years of follow-up in individuals with prediabetes was 0.29%, 3.67%, 11.94% and 19.89%, compared with 0.08%, 0.58%, 5.14% and 9.83% in the quartile at highest risk according to the clinical + PGS model (Fig. 3, ESM Table 8). This showed that individuals considered at high polygenic risk were at about half the absolute risk compared with individuals with prediabetes over a 20 year follow-up period.

## Discussion

This paper reports a systematic study of the predictive value of molecular features selected across several omics layers using a machine-learning approach to develop sparse prediction models and test their performance over and above a currently established standard clinical prediction model for type 2 diabetes, which accounts for participant characteristics such as age, sex, anthropometry and lifestyle, among others. Our results show that a combination of different omic features improved the performance beyond the clinical standard, of which the type 2 diabetes PGS was the major contributor. However, individuals at high predicted polygenic risk were at a substantially lower absolute risk than people with prediabetes, suggesting limited potential value in targeted genetic screening for preventative interventions, if the absolute risk of people with prediabetes is taken as the benchmark against which to determine societal willingness to implement interventions to other subgroups.

Early evidence from randomised controlled trials showed the effectiveness of behavioural and lifestyle interventions to reduce incidence of type 2 diabetes in individuals at high risk [34]. This prompted a substantial amount of research to focus on the development and enhancement of prediction models to enable early detection and intervention in high-risk groups. Beyond well-established patient-derived risk factors, the search for novel biomarkers has led to only marginal improvements with very few successful examples [7]. For example, national screening programmes, such as the NHS Diabetes Prevention Programme (NHS-DPP), have proven to be successful in significantly reducing the population incidence of type 2 diabetes, with up to 23,776 cases prevented in a 1 year period [35], by enabling identification of high-risk individuals using basic patient-derived risk factors and HbA_1c_ measurements.

According to current screening guidelines, individuals with HbA_1c_ above the diabetic or prediabetic threshold would be referred for treatment or preventative behavioural interventions. Therefore, improved predictive strategies would be of most theoretical benefit in subgroups of individuals who are missed by HbA_1c_ screening but who are at high risk of type 2 diabetes and its associated comorbidities and could benefit from preventative interventions that would not otherwise be considered. Here, we identified a subset of people with HbA_1c_ below the prediabetic threshold for whom a combination of ten omic features improved predictive performance beyond the standard clinical model. Since the type 2 diabetes PGS was the strongest contributor to this omic predictive signature, we leveraged genotyping done in the entire EPIC-Norfolk study to estimate the absolute risk in individuals with a high polygenic type 2 diabetes burden. Our results show that over a 20 year follow-up period the absolute risk in individuals at high polygenic risk was less than half that of individuals with prediabetes, therefore highlighting the limited feasibility of implementing targeted genetic screening, as this would translate into a large number needed to treat, and in line with previous studies showing a low absolute risk in lean individuals with a high genetic risk [18]. This is assuming that the relative risk reduction following preventative interventions would be the same in all individuals irrespective of genetic risk. Currently, there is no evidence from trials, such as the NHS-DPP, that this assumption is incorrect [36].

While studies have reported significant associations of omic signatures and incident type 2 diabetes [14, 37, 38], consistent assessment of performance against a clinical gold standard in a formal prediction framework has often been missing. Furthermore, several studies have tested large omic signatures with up to hundreds of proteins or metabolites, which limits their potential to be feasibly translated into clinical settings given the costs and need to validate and harmonise hundreds of assays. We addressed this limitation by developing sparse models, restricting the omic signatures tested to the ten most informative features, and demonstrated limited value of more inclusive signatures.

Among the top ten features selected in each omic layer, we identified novel biomarkers that might reflect specific aetiological processes and organ systems linked to type 2 diabetes risk. Examples include BGLR, PLXB2 and metabolites of unknown structural identity but consistent spectral pattern (X-22822, X-23637, X-12063 and X-11564). PLXB2, a cell surface receptor for semaphorins that has a known role in axon guidance and cell migration [39], has also been shown to be expressed in both pancreatic insulin-producing beta and glucagon-producing alpha cells [40]. This suggests that its presence in plasma might be a marker of pancreatic beta and/or alpha cell damage, a hallmark of early pathways into type 2 diabetes. Furthermore, PLXB2 has been recently found to be putatively causally associated with type 2 diabetes [41] and we have previously found evidence for this protein to be a causal candidate associated with systolic blood pressure [42], suggesting that this protein may represent a potential molecular link between type 2 diabetes risk and cardiovascular comorbidities. Our feature selection approach identified several validated biomarkers that have already been shown to be associated with type 2 diabetes risk, including mannose [43], glycine [44] and IGFBP-1 [45], among others. We further identified the proteins CBPM and serine protease HTRA1, which we have previously shown to be strongly discriminative for impaired glucose tolerance and to be associated with body fat distribution and inflammation [46]. Furthermore, circulating proteins, like ApoF and IGFBP-2, have been associated with non-alcoholic fatty liver disease [47], a risk factor for type 2 diabetes. Our findings therefore suggest that predictive circulating biomarkers may represent early derangements in pathways and organ systems that can be detected in individuals on the path to type 2 diabetes.

While a strength of our study is the comprehensive case ascertainment and virtually complete follow-up among deeply phenotyped participants, several limitations are worth noting. First, our analysis was based on participants of European ancestry, limiting the generalisability of our results across populations of different ethnic backgrounds. To date, few studies [48] have systematically compared type 2 diabetes prediction models across different ancestries, and further work is needed in more ethnically diverse populations. This would be of particular interest in the context of prognosis since susceptibility to specific comorbidities might be far more prevalent in specific ethnic groups. Second, the technology used for proteomics profiling relies on preserved protein structure for recognition by the affinity reagents, meaning that protein-altering variants could lead to biased measurements. However, we have previously shown that LASSO-selected protein candidates were more likely to replicate well across proteomics platforms [46]. Finally, since our study integrated information across several omic layers, this was only available in a relatively small subset of individuals which did not enable estimation of the cumulative type 2 diabetes incidence in individuals predicted to be at high risk by the full omic signature. This further meant that external replication of our results is currently not possible due to the unique depth of information incorporated in these analyses.

In summary, we have shown that the integration across several omics layers provides significant improvements in the prediction of type 2 diabetes over and above what can already be achieved by the current clinical standard. However, when individuals with prediabetes are taken out of the analysis, as they would routinely be offered an intervention, the prediction by a PGS, the most informative omic feature, improves discrimination in individuals who do not have prediabetes but in whom the absolute risk for type 2 diabetes is low. Whether society deems it acceptable to offer preventative interventions to individuals at half the risk of groups for whom interventions are now routinely offered is an economic and political decision. This exemplifies the importance of embedding novel predictive models and biomarkers into the clinical reality to assess and inform the translational potential of innovative strategies.

### Supplementary Information

Below is the link to the electronic supplementary material.

Supplementary file1 (PDF 200 KB)

Supplementary file2 (XLSX 31 KB)

## Data Availability

The EPIC-Norfolk data can be requested by bona fide researchers for specified scientific purposes via the study website (https://www.mrc-epid.cam.ac.uk/research/studies/epic-norfolk/). Data will either be shared through an institutional data sharing agreement or arrangements will be made for analyses to be conducted remotely without the need for data transfer.

## Abbreviations

ALT: Alanine aminotransferase
Apo: Apolipoprotein
AST: Aspartate aminotransferase
BGLR: β-Glucuronidase
CBPM: Carboxypeptidase M
C index: Concordance index
CRP: C-reactive protein
EPIC: European Prospective Investigation into Cancer
GGT: γ-Glutamyl transferase
IGFBP: Insulin-like growth factor binding protein
LASSO: Least absolute shrinkage and selection operator
NHS: UK National Health Service
NHS-DPP: NHS Diabetes Prevention Programme
NRI: Net reclassification improvement
PGS: Polygenic risk score
PLXB2: Plexin B2
