# Supplementary material for: Multi-omic prediction of incident type 2 diabetes
Source: Diabetologia. 2023 Oct 27;67(1):102–12. doi: 10.1007/s00125-023-06027-x (PMC10709231; doi:10.1007/s00125-023-06027-x)

ESM Figure 1. Flowchart of the study. a) Flowchart showing exclusions and study population for the predictive modelling stage of the study. b) Flowchart showing exclusion and final study population to estimate absolute risk in individuals predicted at high risk by the clinical + PGS model.

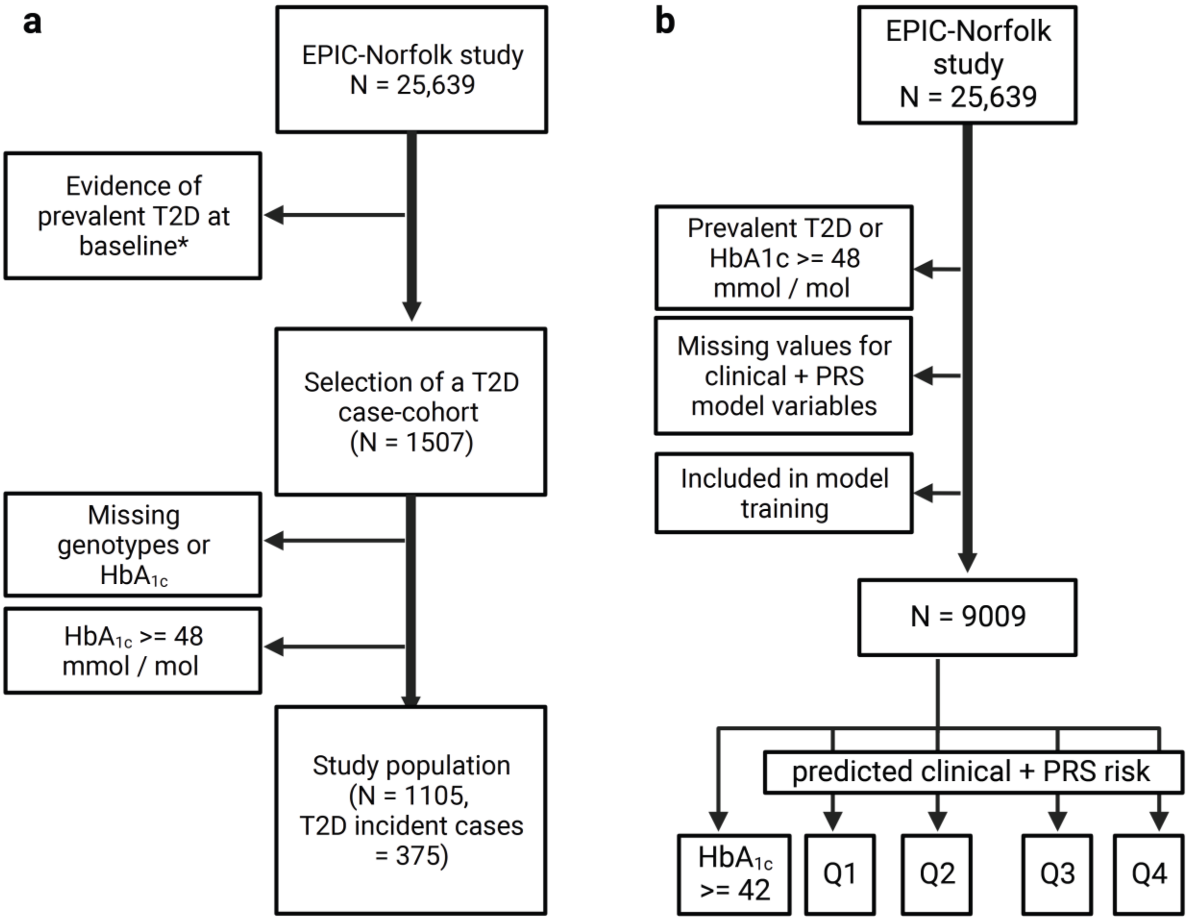

Supplement: Supplementary file 1 — Supplementary file1 (PDF 200 KB) [file 125_2023_6027_MOESM1_ESM.pdf]
